# Molecular detection and quantification of canine parvovirus 2 using a fast and sensitive SYBR^®^ green-based quantitative polymerase chain reaction assay in dogs affected with gastroenteritis

**DOI:** 10.14202/vetworld.2024.2286-2294

**Published:** 2024-10-17

**Authors:** Anthony Loor-Giler, Sara Castillo-Reyes, Silvana Santander-Parra, Martín Campos, Renán Mena-Pérez, Santiago Prado-Chiriboga, Luis Nuñez

**Affiliations:** 1 Laboratorios de Investigación, Dirección general de Investigación, Universidad de las Américas (UDLA), Antigua Vía a Nayón S/N, Quito EC 170124, Ecuador; 2 Facultad de Ingeniería y Ciencias Aplicadas, Carrera de Ingeniería en Biotecnología, Universidad de Las Américas (UDLA), Antigua Vía a Nayón S/N, Quito EC 170124, Ecuador; 3 Facultad de Ciencias de la Salud, Carrera de Medicina Veterinaria, Universidad de Las Américas (UDLA), Quito, Ecuador, Antigua Vía a Nayón S/N, Quito EC 170124, Ecuador; 4 Facultad de Industrias Agropecuarias y Ciencias Ambientales. Carrera agropecuaria. Universidad Po-litécnica Estatal del Carchi (UPEC). Antisana S/N y Av Universitaria, Tulcán EC 040102, Ecuador; 5 Facultad de Ciencias Veterinarias, Universidad Nacional de Rosario (UNR). Boulevard Ovidio Lagos y Ruta 33 Casilda – Santa Fe – Argentina; 6 Facultad de Medicina Veterinaria y Zootecnia, Universidad Central Del Ecuador, Quito, Ecuador, Gatto Sobral y Jerónimo Leiton, Quito EC 170521, Ecuador; 7 Clínica Veterinaria Docente, Universidad de Las Américas (UDLA), Calle Shuara N40-55y Av. De Los Granados, Quito, EC 170503, Ecuador; 8 One Health Research Group, Universidad de Las Américas (UDLA), Antigua Vía a Nayón S/N, Quito EC 170124, Ecuador

**Keywords:** canine parvovirus, gastroenteritis, quantitative polymerase chain reaction, SYBR green

## Abstract

**Background and Aim:**

Viral gastroenteritis in canines is primarily caused by the canine parvovirus 2 (CPV-2). Infections by this virus can cause severe consequences in dogs, such as fever, vomiting, diarrhea, septicemia, systemic inflammation, and immunosuppression. Therefore, the mortality rate of persistent infections caused by this virus is significantly high. The capsid protein VP2 genome of canine parvovirus has undergone many changes, resulting in the emergence of different genotypes, including CPV-2a, CPV-2b, and CPV-2c. Diagnostic procedures often lack the necessary specificity for early infection diagnosis. Early detection of the infection enhances the likelihood of canine survival because the canine will receive prompt therapy. Hence, this study aimed to develop a quantitative polymerase chain reaction (qPCR)-based diagnostic technique using SYBR Green for the rapid and accurate detection and quantification of CPV-2.

**Materials and Methods:**

The assay was specifically designed to identify a portion of the conserved NS gene using primers that amplify a 125-bp fragment. The qPCR method was executed in the fast mode to expedite the process using Power up SYBR Green Master Mix reagent. A standard curve was constructed using the amplified and purified PCR product of the *NS* gene.

**Results:**

The limit of detection and quantification were determined in the one amplified-DNA copy. The standard curve showed an efficiency of 99.5% and inter- and intra-assay coefficients of variation of 0.387%–0.976% and 0.085%–0.430%, respectively. The assay was specific for the amplification of CPV-2, as no amplification was observed for other viral genomes (canine adenovirus II, canine distemper virus, canine coronavirus, and canine astrovirus) or from the negative controls. Inter- and intra-tests for repeatability showed low test variability around the run time. To validate the present assay, 200 samples of fezzes from canines with gastroenteritis and symptoms associated with enteric infection were tested using the qPCR protocol. From the analyzed samples, 136 were positive for CPV-2 by qPCR assay, of which 110 were before diagnostic positive for the virus by endpoint PCR, showing high sensitivity of the current assay. CPV-2 was detected in dogs over 2 weeks old up to dogs 9 years old, where the highest viral concentration found was 16429595 gene copies in dogs aged 2 weeks.

**Conclusion:**

In the present study, a rapid, specific, repeatable, and sensitive assay was developed for the detection and quantification of CPV-2. Furthermore, it was demonstrated that in the population of domestic dogs in Ecuador affected with gastrointestinal disease, the virus is presented in dogs of different ages and not only in young dogs.

## Introduction

Canine parvovirus 2 (CPV-2) is the primary cause of infectious gastroenteritis, with a high incidence and morbidity rate. It can infect both domestic and wild canines. Specifically, unvaccinated puppies are the most susceptible to infection and suffer the most severe signs of the disease [1]. The symptoms of infected dogs range from clinical signs such as watery and bloody diarrhea, inappetence, lethargy, vomiting, and dehydration to death [2].

CPV-2 is a single-stranded negative DNA virus belonging to the genus protoparvovirus and the family Parvoviridae, and it has no envelope [3]. The gene contains approximately 5.12 kb of genetic material with two open reading frames (ORF) translated into four proteins. One reading frame translates for the nonstructural proteins *NS1* and *NS2*, which are responsible for replication, and the second ORF gives rise to the viral capsid proteins *VP1* and *VP2* [2, 4]. The first CPV infection in dogs with gastroenteritis was reported in 1978 and was named CPV-2 to differentiate it from the canine minute virus (CPV-1) [5]. Shortly after its first appearance, the virus carried mutations that caused the CPV-2a variant in 1979 [6]. The second variant, CPV-2b, was first described in 1984 in the United States [7]. Finally, a new variant of CPV-2c was reported in 2001 in Italy [8]. CPV-2 variants have been shown to differentiate by changes at position 426 of the *VP2* protein sequence and are classified as Asn for variant 2a, Asp for variant 2b, and Glu for variant 2c [5]. These variants of the capsid protein have shown direct interaction with cellular receptors and the intensity of infection [9].

After its appearance, CPV-2 spread arduously throughout the world, resulting in high mortality in affected canine populations [4]. CPV-2 capsid proteins recognize intestinal crypts and lymphoid organs. Therefore, vomiting, diarrhea, and anorexia are the most common clinical effects, leading to dehydration and hypovolemic shock [10]. Immunosuppression is a common sign in infected canines and is characterized by a decrease in lymphocytes and breakdown of the intestinal wall [11]. These consequences can give patients access to bacterial or parasitic diseases and further viral infections [12]. Therefore, in patients infected with canine parvovirus, septicemia, systemic inflammation, and clot formation frequently occur, contributing to the severity and mortality of the disease [13].

At present, the original CPV-2 vaccine is available only in commercial vaccines, and variants 2a, 2b, and 2c are distributed in canine populations worldwide [14, 15]. Due to the presence of antigenic variants in the sequence of the *VP2* protein, vaccination often does not provide sufficient protection in canine patients against infection by variants 2a, 2b, and 2c [16]. Early diagnosis of the virus in dogs, especially in puppies, has been shown to ensure the effectiveness of treatments and the probability of survival, reaching 80%–95% survival in infected canines [12]. Conventional diagnostic methods based on enzymatic assays, such as enzyme-linked immunosorbent assay (ELISA) or agglutination tests, are not sufficiently sensitive for virus detection, but they are widely used throughout the clinic [17]. Molecular detection methods such as polymerase chain reaction (PCR) and quantitative PCR (qPCR) have demonstrated high sensitivity for detecting the causative agents of infectious diseases. Therefore, the detection and quantification of CPV-2 via qPCR using Syber Green is a sensitive and efficient alternative for detecting this virus [18, 19]. There is a need to implement cost-efficient methods for the detection of this enteric virus with high sensitivity, specificity, and up-to-date capacity to detect the different genotypes of CPV-2.

Therefore, the objective of this study was to develop a sensitive and rapid diagnostic method to detect and quantify CPV-2 using SYBR^®^ Green-based qPCR targeted to amplify a conserved region of the *N*S1 gene present in all CPV-2 genotypes reported so far, as well as the strains reported in GenBank, validating this capability using confirmed strains of the three CPV-2 genotypes sequenced from canines with gastrointestinal disease identified in Ecuador, given their genetic variability in the VP-2 region.

## Materials and Methods

### Ethical approval

All procedures conducted in the present study were approved by the Committee on the Care and Use of Laboratory and Domestic Animal resources of the Agency of Regulation and Control of Phytosanitary and Animal Health of Ecuador, under the approval serial number #INT/DA/019.

### Study period and location

The study was conducted from February to November 2023 with fecal samples from dogs affected with gastroenteritis from Pichincha Province, Ecuador.

### Sampling and DNA extraction

In the present study, 200 fecal samples from dogs with symptoms of enteric diseases, mainly diarrhea that arrived at the University of the Americas (UDLA) veterinary hospital for veterinary care were used. These samples were subjected to a molecular assay that tested positive for CPV-2 at the molecular level by end-point PCR. The selected samples were rescreened for CPV-2 through molecular analysis using qPCR to detect and quantify the virus, allowing the qPCR assay to be standardized and validated.

A 1:1 suspension of the sample was prepared in a 2 mL microcentrifuge microtube with 1 mL of 1× phosphate-buffered saline (pH 7.4). The suspension consisted of an average of 0.66 g of fecal material. The samples were homogenized, frozen at −80°C for 10 min, thawed in a water bath at 56°C for 1 min, and homogenized. This procedure was repeated 3 times, and the samples were then centrifuged at 12,000× *g* for 20 min. A 200 μL aliquot of the supernatant was placed in a 1.5 mL microcentrifuge microtube and subjected to extraction using the phenol/chloroform method [20]. DNA samples were diluted to 1:10 with ultrapure water before being passed through the PCR protocol.

### Primer design and standard DNA construction

This study used two pairs of primers targeting a conserved region of the NS gene present in all genotypes of CPV-2 (Table-1) [16]. The design of primers used in this study was carried out using the software package Geneious Prime 2022.1.1. (Geneious by Dotmatics, Boston, MA 02110, United States). An alignment was constructed using 150 sequences of CPV-2 and its variants deposited in National Center for Biotechnology Information (https://www.ncbi.nlm.nih.gov/). These sequences were used to choose the primers, ensuring the method’s specificity for the different CPV-2 variants.

**Table-1:** Primers used in this study.

Primer	Gene	Assay	Sequences	Product	Reference
NS-FEXT	NS	PCR	5’- GACCGTTACTGACATTCGCTTC-3’	2254 pb	[16]
NS-REXT			5’- GAAGGGTTAGTTGGTTCTCC-3’		
NS-CPV-DEG-F		qPCR	5’- CGCTTGCACGTCTTTGTGAG-3’	125 pb	This study
NS-CPV-DEG-R			5’- CTCCTCTGACTCCGGACGTA-3’		

CPV=Canine parvovirus, qPCR=Quantitative polymerase chain reaction

For standard curve construction, a CPV-2-positive sample was subjected to endpoint PCR to amplify the complete *NS* gene [21]. The PCR product was subjected to enzymatic purification using ExoSAP-IT Express (Applied Biosystems, Santa Clara, CA 95051 USA) according to the manufacturer’s instructions. The purified amplicon was quantified using Nano Drop equipment (Thermo Fisher Scientific, California, CA, USA). The DNA Copy Number and Dilution Calculator web tool was used to calculate the quantity of recombinant DNA necessary to make the first dilution with a known quantity of DNA copies. Then, 10-fold serial dilutions from 10^9^ copies to 1 copy were prepared to determine the sensitivity and amplification efficiency of the qPCR assay.

### Real-time PCR-qPCR assay

The qPCR reaction was performed using a final volume of 10 μL, 5 μL of Power Up™ SYBR™ Green Master Mix 2×, 0.8 μM of each primer, 1 μL of the DNA and UltraPure™ DNase/RNase-Free Distilled Water dH_2_O (Invitrogen by Thermo Fisher Scientific) necessary to complete 10 μL. The amplification protocol was set up in fast mode under the following conditions: A 2-min cycle at 50°C for enzyme activation, a 2-min cycle at 95°C for initial denaturation, and 40 cycles of 95°C for 3 s of denaturation and 60°C for 30 s for annealing and extension of the DNA template. The melting curve was generated by heating at 95°C for 15 s, followed by lowering the temperature to 60°C for 1 min and heating to 95°C.

The DNA extracted from the 200 fecal samples was subjected to qPCR using the CFX96 Touch Real-Time PCR Detection System (Bio-Rad Laboratories, Inc., Hercules, CA, USA). All samples were run in duplicate, and absolute quantification was performed using the standard curve for each assay. Two non-template controls were placed in each run.

### Limit of detection (LOD) and quantification

The detection and quantification limits were determined using the standard curve. The LOD was defined as the lowest DNA concentration present in the tenfold dilution series detected by the assay, and the limit of quantification (LoQ) was determined as the lowest DNA concentration that the assay could quantify and maintain in the linear portion of the standard curve.

### Repeatability of the assay

To assess the qPCR assay’s intra- and inter-assay repeatability and stability, 10-fold serial dilutions of the reference samples were prepared. According to the test results, the average value of Ct and the coefficient of variation (CV) were calculated, and the assay’s stability was evaluated using CV.

Inter-assay repeatability: Five 10-fold serially diluted reference samples were amplified by qPCR 5 times under the same reaction conditions. Intra-assay repeatability: Five 10-fold serially diluted reference samples were prepared, and five replicates were run for each dilution factor. The quantitative PCR assays were performed simultaneously.

### DNA sequencing and phylogenetic analysis

From the positive tested samples, some were randomly selected, and a 1756-bp fragment of the VP2 complete gene of CPV-2 was amplified using conventional PCR according to a previously described protocol by Rez *et al*. [16]. The PCR product was subjected to enzymatic purification using ExoSAP-IT Express (Applied Biosystems, Thermofisher Scientific) according to the manufacturer’s instructions. The purified PCR product was used for SANGER-type sequencing in the forward and reverse directions using a BigDye^®^ Terminator v3.1 Cycle Sequencing kit (Thermo Fisher Scientific). Sequencing reactions were performed using an ABI 3730 DNA Analyzer (Thermo Fisher Scientific). The primer walking strategy was used to obtain the complete sequence of *VP1* gene. The electropherograms obtained were analyzed and edited using Geneious Prime^®^ 2023.1.1 (Geneious by Dotmatics) program; the ORF finder tool in Geneious Bioinformatics was used to determine the complete CDs of *VP2* gene. The Basic Local Alignment Search tool (https://blast.ncbi.nlm.nih.gov/Blast.cgi) was also used to compare the similarity of each obtained sequence to other CPV2 sequences in GenBank. An alignment was built with the *VP2* obtained sequences and other sequences of different strains of CPV-2 existing in GenBank (https://www.ncbi.nlm.nih.gov/genbank/) using Clustal X 2.1 software (http://www.clustal.org/clustal2/) for the nucleotide (NT) and amino acid (AA) sequences, respectively. Subsequently, phylogenetic analysis with the NT alignment was performed using Molecular Evolutionary Genetics Analysis version 11 (https://www.megasoftware.net/), using a Neighbor-joining statistics method along with a p-distance substitution model and phylogeny test bootstrap model. Three AA sequences were analyzed and classified according to the changes at position 426 of the *VP2* protein sequence into Asn for variant 2a, Asp for variant 2b, and Glu for variant 2c.

### Specificity of the qPCR assay

A specificity assay using nucleus acids extracted from canine adenovirus II (CAV-2), canine distemper virus (CDV), canine coronavirus (CCoV), and canine astrovirus (CaAstV) isolates was performed. In addition, positive samples for each CPV-2 variant (2a, 2b, 2c) previously sequenced as described above were used to verify the assay’s ability to detect the three currently reported variants.

### Statistical analysis

Descriptive statistics were used to evaluate the positivity of the samples for each virus, and the percentage of positive and negative samples was plotted against the information provided on the age of animals. Chi-square analyses were used to determine if age is related to the presence of CPV-2, and a Kruskal–Wallis test was used to determine the degree of significance between the viral load of the different age groups. All analyses were carried out using a significance level of 5%. To compare the results detected by this method with those previously diagnosed, the Kappa coefficient, sensitivity, and positive predictive value (PPV) and negative predictive value (NPV) were calculated. All statistical analyses were performed using the free Jamovi software version 2.3.

### Accession numbers

The NT sequences of the complete *VP2* gene of CPV-2 identified in the present study were deposited in GenBank under the accession numbers UDLA 231 CPV-2a (OQ557584); UDLA 15 CPV-2b (OQ557585), and UDLA 82 CPV-2c (OQ557586).

## Results

### Standard curve determination

**Figure-1:**
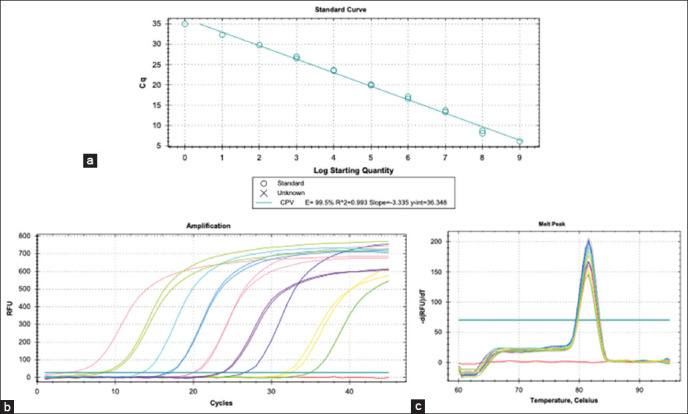
Standard curve of the fast and sensitive quantitative polymerase chain reaction method based on SYBR^®^ Green for canine parvovirus detection and quantification of the conserved region of the nonstructural gene; (a) Standard curve with 10-fold serial dilutions; (b) Amplification plot of the standard curve; (c) Melting curve.

The 10 dilutions generated a standard curve with an efficiency of 99.5%, slope of 3.335, and correlation coefficient of 0.993 (Figure-1a). No dimers were observed during any run.

### LOD and quantification

The standardized method detected up to 10^9^ copies of the DNA (Figure-1a). LoD and LoQ represent one target gene copy (Figures-1a and b). The melting curve showed a single unaltered peak with no alterations and a melting temperature of 81.5°C (Figure-1c). No other peaks were observed. No curves were formed in the non-template control, and no dimers were presented.

### qPCR run time

The running time of the method under fast conditions was approximately 1 h, and with the melting curve, it was approximately one and a half hours. The current assay under standard conditions extends to approximately 2 h without a melting curve. The test under fast conditions showed high sensitivity, amplifying a few to high numbers of DNA gene copies (GC).

### Repeatability of the assay

Repeatability analysis performed with curve dilutions from 10^8^ to 10^4^ copies showed an inter-assay CV of 0.387%–0.976% and an intra-assay CV of 0.085%–0.430% (Table-2).

**Table-2:** Repeatability assays using curve dilutions from 10^8^ to 10^4^ copies of genetic material.

Copy number	Inter-assay		Intra-assay	
	
	Cq mean	Cq SD	Cq mean	Cq SD
10^8^	17.424	0.943	17.965	0.530
10^7^	20.661	0.665	20.932	0.603
10^6^	23.768	0.876	23.20	0.694
10^5^	26.024	0.967	26.21	0.907
10^4^	30.781	0.587	31.18	0.185

SD=Standard deviation

### Specificity and Sensitivity of qPCR

The test performed with isolates of CAV-2, CDV, CCoV, and CaAstV showed no amplification or nonspecific production by other viral causes of gastroenteritis. Samples corresponding to the three genotypes of CPV-2 grouped in the CPV-2 phylogenetic tree (Figure-1) showed amplification in all cases.

### Evaluation of qPCR for CPV detection

**Figure-2:**
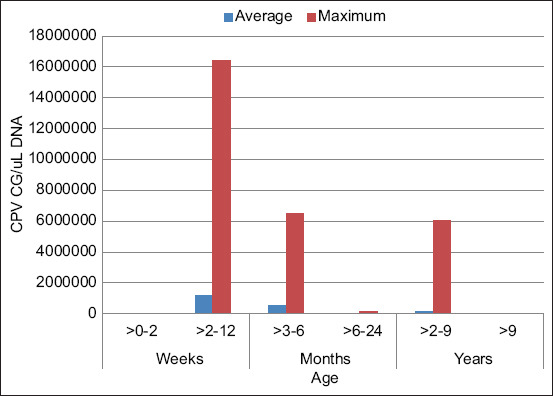
Detection and quantitation of canine parvovirus using sensitive, fast quantitative polymerase chain reaction based on SYBR^®^ Green.

The qPCR based on SYBR Green proposed in this study was able to detect CPV-2 DNA in 136 out of 200 enteric samples submitted to the qPCR assay. As was mentioned above, the presence of CPV-2 was previously identified in some analyzed samples (110/200); however, with the assay proposed in this work, it was possible to identify 26 extra samples that were considered negative for CPV-2. Pre-detection showed 26 samples of false negatives, where the kappa coefficient showed a value of 0.828, so its sensitivity is 0.679, PPV of 1 and NPV of 0.711. The highest viral concentration was 16429595 GC in dogs aged 2 weeks (Table-3). The highest average viral load was 1158490 GC in dogs aged up to 12 weeks. Figure-2 shows that both the average virus detected and the highest load decreased with increasing dog age. The exception was in dogs aged 2–9 years, where the average viral load increased to 116351 GC and the maximum was 6024545 GC. All 136 positive samples exhibited the same melting temperature of 81.5°C. No dimers were presented during any run. Negative controls showed no dimers and no amplification.

**Table-3:** Detection and quantification of canine parvovirus (CPV-2) by qPCR in enteric samples from dogs with gastrointestinal disease symptoms.

Age	Total samples	Positive samples	Average of GC/µL DNA (0.00022 g of fecal material)	Maximum GC/µL DNA	Minimum GC/µL DNA
Weeks					
0–2	200	0/7	-	-	-
>2–12		55/59	1158490	16429595	1
Months					
>3–6		15/19	509351	6519310	1
>6–24		13/24	16808	164050	3
Years					
>2–9		53/87	116351	6024545	1
>9		0/4	-	-	-

GC=Gene copies, qPCR=Quantitative polymerase chain reaction

### Statistical analysis

Chi-square analysis showed that age is related to the presence of CPV-2 (p < 0.001). The Kruskal–Wallis analysis showed that the viral concentration in dogs aged 2–12 months was significant (p < 0.001) in relation to the other age groups where the virus was quantified.

### DNA sequencing and phylogenetic analysis

**Figure-3:**
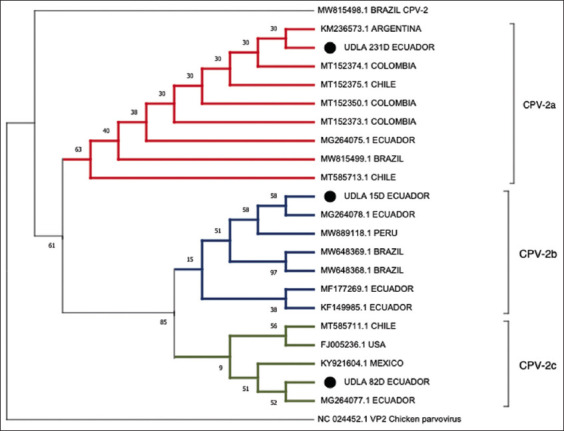
Phylogenetic relationships between the CPV-2 sequences used in the present study for the three genotypes and other canine parvovirus (CPV) sequences from different continents where it is repotted at National Center for Biotechnology Information based on the VP-2 protein sequence. The sequences were aligned using the CLUSTAL W method in ClustalX2 2.1. The phylogenetic tree was constructed using the molecular evolutionary genetics analysis 11 software package. Numbers along the branches are bootstrap values for 1000 replicates. Chicken parvovirus (ChPV) was used as an outgroup and a CPV-2 vaccine corresponding to the original genotype. USA=United States.

Phylogenetic analysis using the three sequences used in this study revealed a clear division of genotypes a, b, and c into three different clades, separating them from the original CPV-2 genotype (Figure-3). Sequences b and c show closeness to previously reported sequences of CPV in Ecuador, whereas the sequence of genotype A is closer to that previously reported in Argentina.

**Figure-4:**
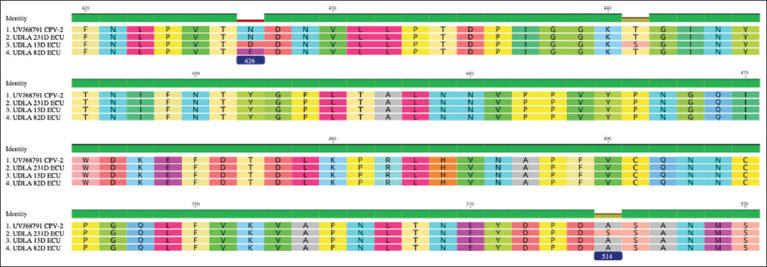
Nucleotide sequences of the VP-2 region of the samples used for genotyping from position 415–529 extracted from Geneious Prime 2023.0.1. 426=Discrimination between Asn (CPV-2/CPV-2a), Asp (CPV-2b), and Glu (CPV-2c). 514=Discrimination between Ala (CPV-2), Ser (CPV-2a).

The sequences show minimal differences in NTs and AAs (Table-4). Position 426 in the AA sequence of the VP-2 protein was used to differentiate genotypes according to AA changes to N (CPV-2 and CPV-2a), D (CPV-2b), and E (CPV-2c), and at position 514 with AAs A (CPV-2) and S (CPV-2a) (Figure-4). An additional AA change was observed at position 440 of sample UDLA 15D from T to S.

**Table-4:** Comparison between nucleotide and amino acid sequences of CPV-2 samples sequenced and genotyped in the present study and the original genotype sequence.

No.	Genotype	Sequence identification	Amino acid identity			

			1	2	3	4
1.	CPV-2	MW815498.1	-	98	98	98
2.	CPV-2a	UDLA 231D	99	-	99	99
3.	CPV-2b	UDLA 15D	98	98	-	99
4.	CPV-2c	UDLA 82D	99	98	99.5	-
			Nucleotide identity			

CPV-2=Canine parvovirus 2, UDLA=University of the Americas

## Discussion

The diagnosis of viruses causing gastroenteric diseases significantly increases the probability of survival in animals [21]. Therefore, developing a sensitive, repeatable, and specific molecular diagnostic method for detecting and quantifying CPV-2 by qPCR based on SYBR Green is presented as a feasible solution to this problem. Commonly, CPV-2 infections are diagnosed using serological methods such as ELISA, rapid CPV antigen and antibody tests, or conventional PCR to amplify fragments of the viral genome [11, 22, 23]. However, the efficiency of these methods is low and specific. In addition, these methods do not allow quantification of patients’ viral loads to determine possible infection status. The use of qPCR allows the quantification of viral particles present in the intestinal tract of canines [23]. Here, it showed a fast qPCR assay based on SYBR Green for the detection and quantification of CPV-2, where the standard curve showed an efficiency of 99.5%, which provided high fidelity of the result and quantification of the sample. The assay presented in this study shows LoD and LoQ in one copy of a specific region of the NS gene from CPV-2. When 200 samples were run with this assay, 136 positive samples were detected and quantified, but these samples initially tested with other molecular assays showed only 110 positive samples. The present method was able to detect and quantify up to one copy of viral DNA in 1 μL of sample corresponding to 0.0002 g of fecal material (Table-2). This result demonstrates that the qPCR assay is more sensitive than commonly used assays [24]. In addition, inter-assay and intra-assay repeatability tests showed CVs of <0.1; thus, the detection assay proposed in this study is considered highly repeatable over time.

The primers used for this method were designed using different sequences of the conserved *NS* gene of different CPV-2 variants. The use of these variant sequences provides high sensitivity for the method, as it can detect all CPV-2 genotypes [25]. Speed in obtaining adequate diagnosis results is essential for decision-making concerning the treatment that patients will receive to save their lives. Thus, the proposed test shows that a shorter time than 1 h is necessary for the detection and quantification of CPV-2 DNA, showing itself to be a valuable tool for diagnostic laboratories and veterinary clinics because end-point PCR molecular assays need several hours to amplify the specific region of CPV-2 DNA, in addition to needing least 1 more h to obtain results through electrophoresis. The speed of the molecular assay presented here will significantly contribute to the rapid diagnosis of CPV-2 infection, guiding veterinarians toward rapid and specific treatment against the infectious agent detected and helping in the care of pets. In some studies, endpoint PCR amplification was inefficient for all samples previously diagnosed with CPV-2 from animals with signs of enteric disease [24, 26].

In Ecuador, CPV-2 was reported exclusively in puppies showing clinical signs of enteric diseases, principally diarrhea. All these reports were carried out using end-point PCR [27, 28], and any of them showed the viral GC present in the samples, nor did we analyze the presence of CPV-2 in animals of different ages. In the present investigation, none of the canine samples from 0 to 2 weeks old presented with viral loads, which may be due to the transfer of antibodies from the mother during lactation in the early stages of life of mammals [29]. However, none of the patients older than 9 years had viral loads. In addition to canines aged 0–2 weeks, this may be due to the low number of samples available from patients at these ages. Samples from canines aged 14 weeks showed the highest average and maximum viral loads among all samples. These high rates of GC may be due to the lack of maturation of lymphoid and gastrointestinal organs mainly affected by CPV-2 and the lack of mature immunity [26]. On the other hand, being the first CPV-2 infection in the patients, given the early age of canines, they do not have antibodies to the variants in circulation at the time of disease [17]. The samples from dogs aged 2 weeks to canines aged 24 months showed a constant decrease in viral load. This may be due to intermittent exposure to variants of the virus, which, if the canine survived, would provide antibodies to fight the disease more effectively [30]. Samples from 2 to 7-year-old trials showed a further increase in the average and maximum viral load in patients related possibly to the aging of the canine species in which, as age increases, they are more susceptible to viral infections associated with poor management and poor nutrition as well as the lack or absence of vaccination against CPV-2 by the carelessness of the owners, which in our country is something daily [31]. The phylogenetic analysis showed that the sequences reported in this article as circulating variants in Ecuador are intimately linked to sequences previously reported in Ecuador Figure-2; with the only exception of the sequence UDLA 231D, which is closer to a sequence from Argentina. This may be due to a migration of the variants through Latin America; however, further studies are needed to affirm or deny this.

Several commercial methods are available for the detection of CPV-2; however, most are based on antigen detection [32], which has shown inefficiency in detecting up to 24% of positive samples. On the other hand, despite the existence of molecular methods based on qPCR, most use hydrolysis probes as fluorescence emitters [33–35], which presents a problem due to the high cost compared with SYBR Green [36, 37]. Ecuador and many countries in South America do not have manufacturers of several reagents used in molecular biology, and importing these products brings high economic costs; for this reason, saving any reagent makes a difference when developing and implementing pathogen detection assays. In the case of the proposed diagnostic method, hydrolysis probes are not used, substantially reducing the cost of the molecular test without decreasing the sensitivity and effectiveness for detecting and quantifying CPV-2. Finally, although a SYBR Green-based method has been described for the detection of this pathogen [38], it has a lower detection limit than that of the proposed assay (LoD = 10 copies) and does not produce positive results for quantification below 1000 copies.

## Conclusion

The present study demonstrated the high sensitivity, repeatability, and specificity of a rapid qPCR diagnostic method for the detection and quantification of CPV-2 in dogs of various ages. Presenting the ability to identify all genotypes and strains of this virus reported to date. Furthermore, the cost-effectiveness of SYBR Green as a method for detecting and quantifying CPV-2 makes it a financially viable option for widespread diagnosis. Due to limitations such as the low number of samples, non-epidemiological sampling, limited sequences, and SANGER sequencing instead of NGS, it was not possible to conduct more specialized analyses of the epidemiology and molecular characteristics of the virus. In the future, it will be necessary to conduct epidemiological studies that consider the possible epidemiological events of CPV-2 and its genotypes, coinfections, and potential mutations, including whole-genome sequencing, to identify sequence modifications that could alter the behavior of this pathogen.

## Authors’ Contributions

ALG: Conducted the study and wrote the original manuscript. SCR: Execution and analysis of the study. SSP: Execution of the study and revision of the manuscript. MC, RMP, and SPC: Draft revision and sample collection. LN: Project design, planning, and drafted and revised the manuscript. All authors have read and approved the final manuscript.

## References

[1] Elbaz E, El-Tholoth M, Abo Elfadl E.A, Mosad S.M (2021). Molecular investigation on the presence of canine parvovirus in Egypt. Comp. Immunol. Microbiol. Infect. Dis.

[2] Goddard A, Leisewitz A.L (2010). Canine parvovirus. Vet. Clin. North Am. Small Anim. Pract.

[3] Miranda C, Thompson G (2016). Canine parvovirus:The worldwide occurrence of antigenic variants. J. Gen. Virol.

[4] Luna Espinoza L.R, Carhuaricra Huamán D, Quino Quispe R, Rosadio Alcántara R.H, Maturrano Hernández A.L (2022). Carnivore protoparvovirus 1 in Peruvian dogs:Temporal/geographical and evolutionary dynamics of virus. Infect. Genet. Evol.

[5] Giraldo-Ramirez S, Rendon-Marin S, Ruiz-Saenz J (2020). Phylogenetic, evolutionary and structural analysis of canine parvovirus (CPV-2) antigenic variants circulating in Colombia. Viruses.

[6] Buonavoglia C, Martella V, Pratella A, Tempesta M, Cavalli A, Buonavoglia D, Bozzo G, Elia G, Decaro N, Carmichael L (2001). Evidence for evolution of canine parvovirus type 2 in Italy. World J. Virol.

[7] Allison A.B, Kohler D.J, Ortega A, Hoover E.A, Grove D.M, Holmes E.C, Parrish C.R (2014). Host-specific parvovirus evolution in nature is recapitulated by *in vitro* adaptation to different carnivore species. PLoS Pathog.

[8] Oosthuizen A, Brettschneider H, Dalton D.L, Du Plessis E.C, Jansen R, Kotze A, Mitchell E.P (2019). Canine parvovirus detected from a serval (*Leptailurus* serval) in South Africa. J. S. Afr. Vet. Assoc.

[9] Franzo G, Tucciarone C.M, Casagrande S, Caldin M, Cortey M, Furlanello T, Legnardi M, Cecchinato M, Drigo M (2019). Canine parvovirus (CPV) phylogeny is associated with disease severity. Sci. Rep.

[10] Hao X, He Y, Wang C, Xiao W, Liu R, Xiao X, Zhou P, Li S (2020). The increasing prevalence of CPV-2c in domestic dogs in China. PeerJ.

[11] Meggiolaro M.N, Ly A, Rysnik-Steck B, Silva C, Zhang J, Higgins D.P, Muscatello G, Norris J.M, Krockenberger M, Šlapeta J (2017). MT-PCR panel detection of canine parvovirus (CPV-2):Vaccine and wild-type CPV-2 can be difficult to differentiate in canine diagnostic fecal samples. Mol. Cell. Probes.

[12] Kelman M, Barrs V.R, Norris J.M, Ward M.P (2020). Canine parvovirus prevention and prevalence:Veterinarian perceptions and behaviors. Prev. Vet. Med.

[13] Kelman M, Norris J.M, Barrs V.R, Ward M.P (2020). A history of canine parvovirus in Australia:What can we learn?. Aust. Vet. J.

[14] Grecco S, Iraola G, Decaro N, Alfieri A, Alfieri A, Gallo Calderón M, Da Silva A.P, Name D, Aldaz J, Calleros L, Marandino A, Tomás G, Maya L, Francia L, Panzera Y, Pérez R (2018). Inter- and intracontinental migrations and local differentiation have shaped the contemporary epidemiological landscape of canine parvovirus in South America. Virus Evol.

[15] Alves F, Prata S, Nunes T, Gomes J, Aguiar S, Aires Da Silva F, Tavares L, Almeida V, Gil S (2020). Canine parvovirus:A predicting canine model for sepsis. BMC Vet. Res.

[16] Rez R.P, Calleros L, Marandino A, Sarute N, Iraola G, Grecco S, Blanc H, Vignuzzi M, Isakov O, Shomron N, Carrau L, Hernández M, Francia L, Sosa K, Tomás G, Panzera Y (2014). Phylogenetic and genome-wide deep-sequencing analyses of Canine parvovirus reveal co-infection with field variants and emergence of a recent recombinant strain. PLoS One.

[17] Aldaz J, García-Díaz J, Calleros L, Sosa K, Iraola G, Marandino A, Hernández M, Panzera Y, Pérez R (2013). High local genetic diversity of canine parvovirus from Ecuador. Vet. Microbiol.

[18] Nuñez L.F, Santander-Parra S.H, Chaible L, De la Torre D.I, Buim M.R, Murakami A, Dagli M.L.Z, Astolfi-Ferreira C.S, Ferreira A.J.P (2018). Development of a sensitive real-time Fast-qPCR Based on SYBR®green for detection and quantification of Chicken Parvovirus (ChPV). Vet. Sci.

[19] Beaver-Kanuya E, Harper S.J (2020). Development of RT-qPCR assays for the detection of three latent viruses of pome. J. Virol. Methods.

[20] Green M.R, Sambrook J (2017). Isolation of high-molecular-weight DNA using organic solvents. Cold Spring Harb. Protoc.

[21] Horiuchi M, Goto H, Ishiguro N, Shinagawa M (1994). Mapping of determinants of the host range for canine cells in the genome of canine parvovirus using canine parvovirus/mink enteritis virus chimeric viruses. J. Gen. Virol.

[22] Santander Parra S, Nunez L, Buim M.R, Astolfi-Ferreira C.S, Piantino Ferreira A.J (2018). Development of a qPCR for the detection of infectious laryngotracheitis virus (ILTV) based on the gE gene. Br. Poult. Sci.

[23] Jiang H, Yu Y, Yang R, Zhang S, Wang D, Jiang Y, Yang W, Huang H, Shi C, Ye L, Yang G, Wang J, Wang C (2021). Detection and molecular epidemiology of canine parvovirus type 2 (CPV-2) circulating in Jilin Province, Northeast China. Comp. Immunol. Microbiol. Infect. Dis.

[24] Alam S, Chowdhury Q.M.M.K, Roy S, Chowdhury M.S.R, Hasan M, Al Mamun M, Uddin M.B, Hossain M.M, Rahman M.M, Rahman M.M (2021). Molecular detection and phylogenetic analysis of Canine Parvovirus (CPV) in diarrhoeic pet dogs in Bangladesh. Vet. Anim. Sci.

[25] Qi S, Zhao J, Guo D, Sun D (2020). A mini-review on the epidemiology of canine parvovirus in China. Front. Vet. Sci.

[26] Decaro N, Buonavoglia C (2012). Canine parvovirus--a review of epidemiological and diagnostic aspects, with emphasis on type 2c. Vet. Microbiol.

[27] Deng X, Zhang J, Su J, Liu H, Cong Y, Zhang L, Zhang K, Shi N, Lu R, Yan X (2018). A multiplex PCR method for the simultaneous detection of three viruses associated with canine viral enteric infections. Arch. Virol.

[28] De la Torre D, Mafla E, Puga B, Erazo L, Astolfi-Ferreira C, Ferreira A.P (2018). Molecular characterization of canine parvovirus variants (CPV-2a, CPV-2b, and CPV-2c) based on the VP2 gene in affected domestic dogs in Ecuador. Vet. World.

[29] Chastant S, Mila H (2019). Passive immune transfer in puppies. Anim. Reprod. Sci.

[30] Mazzaferro E.M (2020). Update on canine parvoviral enteritis. Vet. Clin. North Am. Small Anim. Pract.

[31] Decaro N, Buonavoglia C, Barrs V.R (2020). Canine parvovirus vaccination and immunisation failures:Are we far from disease eradication?. Vet. Microbiol.

[32] Walter-Weingärtner J, Bergmann M, Weber K, Truyen U, Muresan C, Hartmann K (2021). Comparison of eight commercially available faecal point-of-care tests for detection of canine parvovirus antigen. Viruses.

[33] Wang R, Zhang W, Ye R, Pan Z, Li G, Su S (2020). One-step multiplex TaqMan probe-based method for real-time PCR detection of four canine diarrhea viruses. Mol. Cell. Probes.

[34] Yip H.Y.E, Peaston A, Woolford L, Khuu S.J, Wallace G, Kumar R.S, Patel K, Ahani Azari A, Akbarzadeh M, Sharifian M, Amanollahi R, Jafari Jozani R, Khabiri A, Hemmatzadeh F (2020). Diagnostic challenges in canine parvovirus 2c in vaccine failure cases. Viruses.

[35] Streck A.F, Rüster D, Truyen U, Homeier T (2013). An updated TaqMan real-time PCR for canine and feline parvoviruses. J. Virol. Methods.

[36] Tajadini M, Panjehpour M, Javanmard S (2014). Comparison of SYBR Green and TaqMan methods in quantitative real-time polymerase chain reaction analysis of four adenosine receptor subtypes. Adv. Biomed. Res.

[37] Ma L, Zeng F, Cong F, Huang B, Huang R, Ma J, Guo P (2019). Development of a SYBR green-based real-time RT-PCR assay for rapid detection of the emerging swine acute diarrhea syndrome coronavirus. J. Virol. Methods.

[38] Kumar M, Nandi S (2010). Development of a SYBR Green based real-time PCR assay for detection and quantitation of canine parvovirus in faecal samples. J. Virol. Methods.

